# Energy expenditure changes after Roux-en-Y Gastric Bypass

**DOI:** 10.1186/1758-5996-7-S1-A240

**Published:** 2015-11-11

**Authors:** Milene Moehlecke, Manoel Roberto Maciel Trindade, Ana Carolina Mazzuca, Carina Andriatta Blume, Jakeline Rheinheimer, Cristiane Bauermann Leitão

**Affiliations:** 1 UFRGS, Porto Alegre, Brazil

## Background

Weight loss usually decreases energy expenditure (EE) because of changes in body composition (BC). The reduction in EE may contribute, in part, to long-term weight regain. Patients undergoing bariatric surgery might experience a decrease in EE, mainly due to reduced resting metabolic rate (RMR), explained by a decreased lean body mass (LBM), similarly to what occurs to patients after diet-induced weight loss.

## Objective

To assess the effects of Roux-en-Y Gastric Bypass (RYGB) on RMR and BC in severe obese patients after RYGB.

## Materials and methods

This is a prospective cohort study with 28 patients who have undergone RYGB. RMR was assessed prior to surgery and 6 months postoperative by indirect calorimetry (IC). BC was measured at these same time-points using dual-energy X-ray. RMR was adjusted for changes in body weight (BW), i.e., kilocalories per kilogram, and in free fat mass (FFM).

## Results

**Figure 1:**
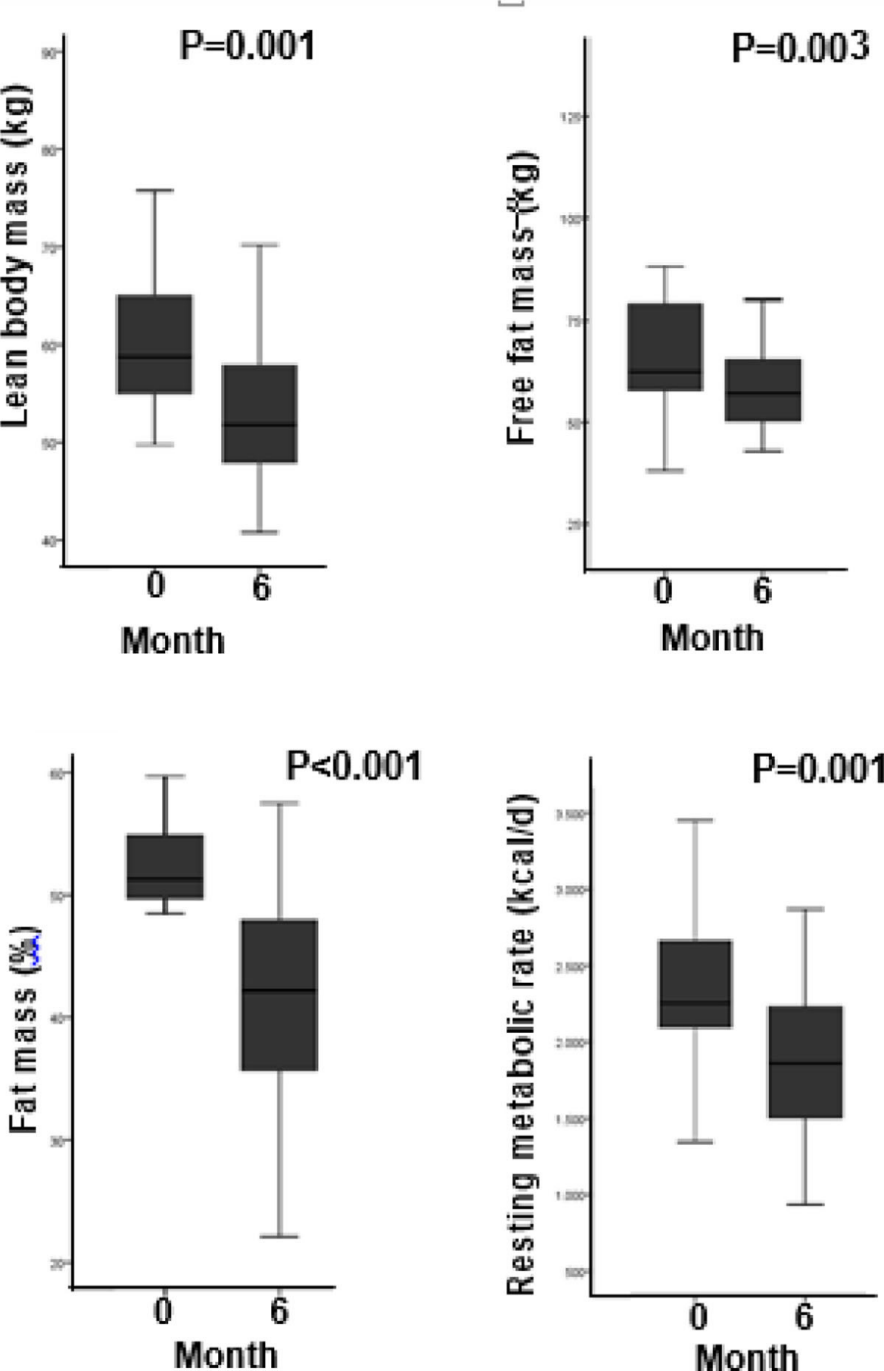
Changes in lean body mass, free fat mass, fat mass and RMR before and after RYGB.

Twenty-two female and 6 male RYGB patients had complete data at baseline and at 6 months, with a mean age of 42±11 yrs., a mean body mass index (BMI) of 49±9 Kg/m^2^ and a mean BW of 128±19 Kg, half of which composed by fat mass (FM) (50±5%). The mean RMR was 2218±595 Kcal/day. Baseline RMR correlated with FFM (r=0.635; P=0.001) (Figure 1); therefore FFM explained about 40% of the variance of RMR. The coefficient of variation (CV) of RMR was 20.8%. The correction of RMR by FFM reduced the CV to approximately 14%. At 6 months, the percentage of excess weight loss was 46±12%. The FM decreased significantly (19±5%, P<0.001), as well as FFM (17±16%, P=0.003), and RMR (-437±504 kcal, P=0.001; Figure 2). The BW-adjusted RMR was unchanged post-RYGB (P=0.223). RMR adjusted for BW was negatively correlated to the total percentage of body fat preoperatively (r=−0.549; P=0.028).

## Conclusion

Weight loss following RYGB Results in FM as well as LBM reduction, which lead to decrease RMR. Such decrease in RMR may limit weight loss over time and even favor weight regain.

